# HIV and NCDs: inevitable interaction in resource limited settings

**DOI:** 10.4314/ahs.v17i4.1

**Published:** 2017-12

**Authors:** James K Tumwine

As the incidence of infectious diseases in LMICs takes a back seat, thanks to the effective vaccines and other public health measures, the importance of non-communicable diseases (NCDs) has come to the fore.^1^ It is in line with this that we highlight HIV and NCDs in Africa as the main theme of this December 2017 issue of AHS.

## HIV/AIDS and other infections

Nalukwago and others from Uganda^2^ are the authors of our lead article in this issue. They sought to establish the effect of interrupted ART on reconstitution of CD4 and CD8 subsets in TB patients. They found that there was a significant increase of naive CD8^+^ cells and a decrease in effector CD8^+^ cells to values near the baseline. They concluded that interrupting ART alters CD8^+^, but not CD4^+^ subsets in patients with less advanced HIV infection and TB.

Namara-Lugolobi and others, also from Uganda, studied factors associated with unknown HIV status among women delivering in Uganda's national and teaching hospital in Kampala.^3^ They found that only 3% of women in labour had an unknown HIV result. Attending ANC and being counselled for HIV testing were associated with having a known HIV status.

Still with the infectious disease theme, we have work on HIV/hepatitis coinfection,^4^ client type and condom use for HIV, and prevention in Kenya.^5^

We have an interesting paper on angio-converting enzyme insertion/deletion polymorphism and susceptibility to Kawasaki disease,^6^ and the *mecA* gene among *staphylococci* from clinical samples in Nigeria.^7^ Still in line with the microbiology interest, Chinese authors have written for us a beautiful article on evaluation and improvement of LAMP assays for analysis of *Escherichia coli* serogroups. They assert that LAMP assays are highly susceptible to non-specific amplication by primer divers.^8^

Does anyone worry about hepatitis E? Well, you should! One in every 8 pig abattoir worker in Kampala tested positive for hepatitis E. This in a country with a hepatitis E pandemic in the Karamoja region.^9^

We end this infectious disease treatise with an interesting study on trypanosomiasis in Kenya. Maina found that rats infected with *Trypanosoma brucei brucei* had abnormal ACTH concentration and histological changes in the pituitary gland.^10^

## Now to non-communicable diseases (NCDs)

Cancer:

Chinese scientists report on cases of breast cancer patients with metastases to the thyroid^11^ while Nwadike and others have written on mammographic classification of breast lesions among women in Nigeria.^12^ The cancer treatise ends with a study of gastrointestinal malignancies in 5 regional referral hospitals in Uganda.^13^ They found that oesophageal, liver, stomach and colorectal cancers are common with increasing trends.

Obesity is a serious NCD. Saudi scientists studied genetic polymorphism of FTO, and found an association between FTO genotype with increasing weight, BMI and leptin.^14^ On the other hand, Nigeria and US scientists studied the CVD risk factors in adult outpatients in rural urban health facilities in Nigeria. They found CVD risk factors highly prevalent. Many were not yet diagnosed and unaware.^15^

A unique study from Mauritius sheds light on oral dysbacteriosis in type 2 diabetes and its role in the progression to cardiovascular disease^16^ while Nigerian authors report on QTC prolongation in black diabetic subjects with cardiac autonomic neuropathy.^17^

*Now NCDs and the environment:* An interesting study from India reports on outdoor aeroallergen and CD14 C(-159) T polymorphism in asthma severity in Kolkata,^18^ while Nigerian authors^19^ report on factors associated with tobacco smoking among long distance drivers in Lagos.

## Growth and development

Abnormalities of the external genitalia and groin are common in Nigeria affecting over a third of boys in primary school.^20^ This report is followed by Odiit's letter on childhood kidney diseases in Uganda,^21^ and a South African report on childhood idiopathic nephrotic syndrome.^22^

It is tempered by a Tunisian study on the long term effect of a school based intervention to prevent chronic diseases.^23^ Then follows a case study of the reality of everyday communication for a deaf child, using sign language.^24^ The child health theme continues with a South African paper on astigmatism,^25^ followed by one highlighting suicidal ideation among adolescents in Swaziland.^26^ The section ends with intestinal candidiasis and antibiotics in Nsukka, Nigeria.^27^

*Health system issues:* The issue of universal health coverage is in vogue. In line with this, Zambian and South African authors assessed regional variations in the effect of removal of user charges on facility based deliveries in Zambia.^28^ Still in South Africa, Oosthuizen and others report on market dynamics of selective serotonin re-uptake inhibitors, in the private sector.^29^

We conclude this health systems section with a piece on the *“Physician's Pledge”*, a sort of dot.com version of the famous *Hippocratic oath* taken by doctors on graduation.^30^ So relevant to ongoing discussions on the ethics of medical practice in a continent with endemic corruption and poorly funded public health services.

The rest of the papers are on erythrocyte ATPase activity, 31 neurocalcin-delta,^32^ corneal macular retinal nerve fiber,^33^ and middle ear malfunction in the elderly.^34^ In addition, we have papers on bowel injury following gynaecological laparoscopic surgery,^35^ and erroneous opinion on cause of death.^36^

This has given us a glimpse into what the AHS team has prepared in this 4^th^ issue of AHS in 2017: NCDs and infections.
